# Rheumatism in Childhood

**Published:** 1904-09-10

**Authors:** 


### RHEUMATISM IN CHILDHOOD.

While everyone is agreed that the manifestations of rheumatism in children are not limited to chorea and arthritis, there is considerable difference of opinion as to the number of ailments which ought rightly to be designated rheumatic. Dr. James Burnet[1] takes rather a comprehensive view of the subject. He thinks that pharyngitis and tonsillitis should be more frequently regarded as rheumatic than they are at present; and he states that in some cases where sore throat is the chief or sole complaint careful light percussion of the heart shows it to be somewhat dilated, while judicious questioning may elicit the complaint of general soreness all over the body. Many of these cases yield rapidly to anti-rheumatic treatment, and the patient should be kept in bed until all doubts about the nature of the case have been dispelled. He also thinks that pneumonia may sometimes be a manifestation of rheumatism, and refers to a case where a child developed chorea shortly after an attack of pneumonia. Psoriasis also may have a direct connection with rheumatism for it most commonly develops in rheumatic subjects and is benefited by anti-rheumatic remedies. Appendicitis, he thinks, is undoubtedly a rheumatic affection in not a few cases, and it is moreover one which is frequently unrecognised. Sometimes the symptoms are clearly attributable to the appendix, but in others there are merely recurrent attacks of abdominal pain relieved by simple treatment. Pain in the side or in the upper part of the chest is a common complaint in childhood and is often quickly relieved by anti-rheumatic remedies. Lastly, children of rheumatic heredity do not always present any definite manifestation of the disease.

They are often, however, extremely nervous and irritable, very sensitive, thin and spare, subject to fits of violent temper, and are generally of a restless disposition and constantly on the look-out for some fresh form of amusement. The nervous restlessness in such cases is liable to increase gradually until there is definite chorea.

With regard to treatment, the best results are obtained from the administration of aspirin or of salicylate of sodium; and children tolerate these drugs better than is generally supposed. To a child of five 10 grains of either remedy may be given every four hours for several days. Another drug which is useful is calomel. It is best administered in doses of half to one grain three times a day. Cardiac conditions are best treated locally by means of an ice-bag, which should be carefully wrapped up in flannel, and also prevented from unduly pressing upon the chest wall. If convalescence is retarded, the patient should be put on small doses of cod-liver oil and the administration of anti-rheumatic remedies continued.

[1] Brit. Jour. Chil. Dis., September.

